# Closed system vacuum assisted administration of high dose radio iodine to cancer thyroid patients: NIMS techniqe

**DOI:** 10.4103/0972-3919.63601

**Published:** 2010

**Authors:** VVS Prabhakar Rao, Pushpalatha Sudhakar, V Kumara Swamy, G Pradeep, N Venugopal

**Affiliations:** Department of Nuclear Medicine, Nizams Institute of Medical Sciences, Hyderabad, India

## INTRODUCTION

There is a necessity for the containment of radiation exposure to the personnel administering high doses of radio iodine solution to cancer thyroid patients. A novel methodology has been devised to effectively administer high doses of radio iodine to patients. This technique is less time consuming, exposure to the person administering the dose is far less, and yet the procedure is simple and patient friendly.

## CONVENTIONAL METHOD

Conventionally radio iodine is administered by directly sucking with a paper straw, from an opened radioactive vial.

**Figure 1:**
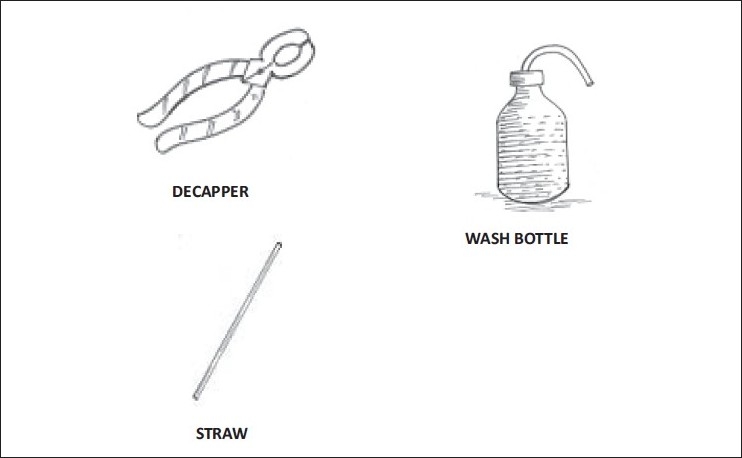
Items required for conventional administration of radio iodine

**Figure 2:**
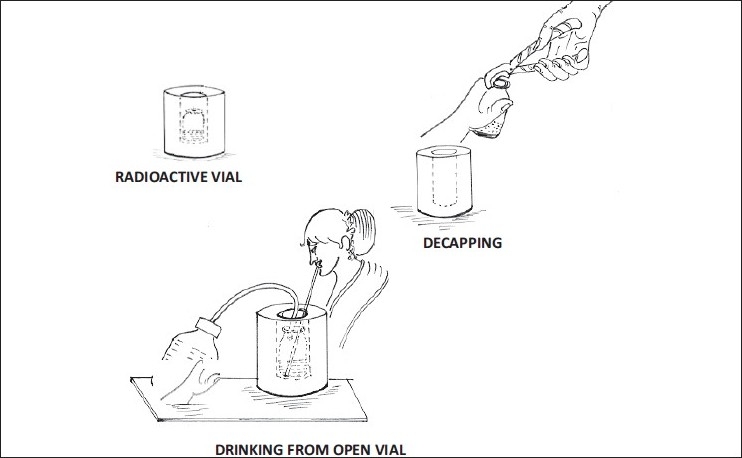
Process of conventional administration of radio iodine

1. The first step involves decapping of the vial containing high-dose radio iodine with a decapper. This requires lifting the vial out of the lead container [Figure 1].
2. The second step involves insertion of a paper straw into the decapped vial, after which the patient is instructed to drink the solution as the technologist keeps pouring plain water into the vial [Figure 2].
3. The emptied radioactive vial, the decapped rubber stopper, and the paper straw will be discarded as loose radioactive waste.

## CLOSED SYSTEM VACUUM-ASSISTED: NIMS TECHNIQUE

**Figure 3:**
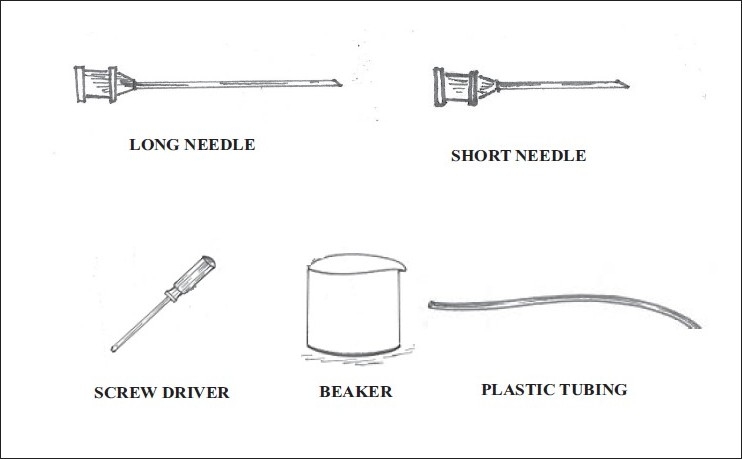
Items required for closed system administration of radio iodine

Materials required: IV sets of plastic tubes, two IV drip needles, one long and one short, and a beaker of water (100 – 200 ml). The plastic tubing is cut into lengths of 12 and 24 inches and fixed to the respective short and long needles [Figure 3].

**Figure 4:**
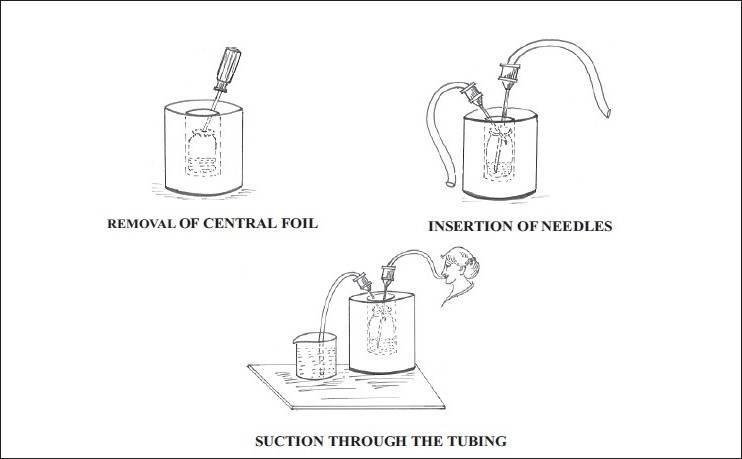
Process of closed system vacuum assisted administration of radio iodine

Procedure: The consignment of the radioactive vial along with the lead pot is placed inside the Fume hood for administration of I-131 solution to the patient [Figure 4].

*Step 1*. The lead pot cover is removed and without lifting the radioactive vial out of the lead pot, the small central aluminum foil covering the rubber stopper is flipped off with the tip of a long metal screwdriver thus exposing the rubber cap.

*Step 2*. The needle with the attached short length plastic tubing is inserted through the rubber cap into the vial only till the top of the vial, the other end of the plastic tubing is immersed in the beaker containing plain water.

*Step 3*. The long needle with the attached longer plastic tubing is inserted into the vial till the tip of the needle touches the bottom of the vial and the free end of the plastic tubing is kept in the mouth of the patient, who is then asked to start sucking slowly and steadily.

## PRINCIPLE OF THE PROCEDURE

As the solution from the sealed vial is sucked, vacuum is created inside the vial, as a result water from the beaker will enter the vial through the short tube flushing the entire radioactive solution as the patient continuously drinks the solution. A quantity of 100 ml of water in the beaker is sufficient to flush the entire solution from the vial leaving no significant activity in the vial. The whole procedure takes only a few minutes.

The procedure is explained to the patient and multiple cold practice runs can be given, which will reduce the patients' apprehension, and will make them drink the solution all by themselves without spillage or regurgitation.

## DRAWBACKS OF THE CONVENTIONAL METHOD

The conventional procedure is more time consuming, and cumbersome, as it requires decapping, and the vial having to be taken out of the lead pot thus exposing the technologist to radiation all the time. Chances of inhalation of the volatile radioactive iodine solution from the opened vial are high. There is a possibility of spillage and contamination if the patient regurgitates or chokes when drinking. The technologist has to be standing next to the patient pouring water into the vial, during the whole process of the patient's drinking it. Radioactive waste is more and in a loose form, consisting of a rubber stopper, opened vial, and the straw.

## ADVANTAGES OF THE CLOSED SYSTEM VACUUM-ASSISTED METHOD

1. Easy and convenient to the patient as well as to the technologist.
2. Radioactive vial containing high-dose iodine is always inside the lead pot without being lifted for decapping.
3. As the vial remains always sealed no volatile radioactive waste is generated.
4. The technologist can monitor the patient from a distance after inserting the needles, instead of standing next to him pouring water into the vial.
5. Minimum radiation exposure to the technologist, due to less time spent and more distance from the patient.
6. No chance of spillage or contamination due to less chances of regurgitation or choking by the patient.
7. Radioactive waste is reduced to a single sealed vial instead of an open vial, rubber cap, and paper straw in the conventional technique.
8. Left over activity in the vial after dose administration is negligible (few micro Curie only).

## CONCLUSION

The newer method has a distinct advantage in terms of ease of administration, reduced radiation exposure to the technologist, generation of less and sealed radioactive waste, and most importantly it is patient friendly. Its implementation in high-dose radio iodine administration will immensely benefit the nuclear medicine personnel.

